# Identification of Regulatory Host Genes Involved in Sigma Virus Replication Using RNAi Knockdown in *Drosophila*

**DOI:** 10.3390/insects10100339

**Published:** 2019-10-11

**Authors:** Jen-Fu Liao, Carol-P Wu, Cheng-Kang Tang, Chi-Wei Tsai, Lenka Rouhová, Yueh-Lung Wu

**Affiliations:** 1 Department of Entomology, National Taiwan University, Taipei 106, Taiwan; m53220492002@livemail.tw (J.-F.L.); funnimax@yahoo.com (C.-P.W.); tom48836064@gmail.com (C.-K.T.);; 2 Czech Academy of Sciences, Biology Centre, Institute of Entomology, České Budějovice 37005, Czech Republic; 3 Faculty of Science, University of South Bohemia, České Budějovice 37005, Czech Republic

**Keywords:** sigma virus, JAK-STAT pathway, IMD pathway, RNA interference

## Abstract

The *Drosophila melanogaster* sigma virus, a member of the *Rhabdoviridae* family, specifically propagates itself in *D. melanogaster*. It contains six genes in the order of 3′-*N*–*P*–*X*–*M*–*G*–*L*-5′. The sigma virus is the only arthropod-specific virus of the *Rhabdoviridae* family. Sigma-virus-infected *Drosophila* may suffer from irreversible paralysis when exposed to a high CO_2_ concentration, but generally, no other symptoms are reported. A recent study reported that host gene expression in immune pathways was not changed in sigma-virus-infected *Drosophila*, which does not necessarily suggest that they are not involved in virus–host interactions. The present study aimed to identify host genes associated with sigma virus replication. Immune pathways JAK-STAT and IMD were selected for detailed study. The results showed that the genome copy number of the sigma virus increased after knocking down the immune pathway genes *domeless* and *PGRP-LC* in *Drosophila* S2 cells. The knocking down of *domeless* and *PGRP-LC* significantly up-regulated the expression of the *L* gene compared to the other viral genes. We propose that the immune pathways respond to sigma virus infection by altering *L* expression, hence suppressing viral replication. This effect was further tested in vivo, when *D. melanogaster* individuals injected with ds*dome* and ds*PGRP-LC* showed not only an increase in sigma virus copy number, but also a reduced survival rate when treated with CO_2_. Our study proved that host immunity influences viral replication, even in persistent infection. Knocking down the key components of the immune process deactivates immune controls, thus facilitating viral expression and replication. We propose that the immunity system of *D. melanogaster* regulates the replication of the sigma virus by affecting the *L* gene expression. Studies have shown minimal host–virus interaction in persistent infection. However, our study demonstrated that the immunity continued to affect viral replication even in persistent infection because knocking down the key components of the immune process disabled the relevant immune controls and facilitated viral expression and replication.

## 1. Introduction

Recent studies have used *Drosophila melanogaster* as a model for examining immune responses to viral infection, including responses to positive-sense RNA virus (*Drosophila* C virus, DCV) and negative-sense RNA virus (sigma virus) invasion [1,2]. The results showed that these viruses produced different symptoms depending on their host. Sigma virus is vertically transmitted and, in endemic areas, infects *D. melanogaster*, making it the only arthropod-specific member of the *Rhabdoviridae* family [2,3]. The RNA genome is similar to that described in *Vesicular stomatitis Indiana*, which comprises the genes *N*, *P*, *M*, *G*, and *L* (in order from 3′ to 5′); however, sigma virus differs due to the presence of the *X* gene between *P* and *M* (i.e., 3′-*N*–*P*–*X*–*M*–*G*–*L*-5′) [3], which is not typically observed in the rhabdovirus genome. Available information on the *X* gene is limited; it is only known to include a conserved region of reverse transcriptase. *G* and *M* produce structural proteins that are embedded in the lipid bilayer. *N* recognizes the viral RNA, and the N-RNA template binds to the RNA polymerase *L* (which is carried within the virions [4]) via the phosphoprotein P [5], forming a ribonucleoprotein complex that is released into the cytoplasm and initiates the processes of transcription and replication upon infection. In its natural course it does not produce marked symptoms [6], although it does cause irreversible paralysis in the presence of high CO_2_ concentrations [7,8]. Furthermore, it does not cause any immune response [9]. Despite this, it does have pathogenic effects (i.e., lower egg viability), which are strongly exhibited when infected female flies mate with uninfected males from a different population [10]. This observation suggests that the infected host can selectively control viral genome expression and subsequently viral replication.

The *Drosophila* model has facilitated the identification of numerous host genes up-regulated in response to pathogen infection. Defense of the host against viruses can be classified into inducible antiviral immunity and RNA interference (RNAi) [11]. To date, a number of major antiviral immune pathways, including Toll, Janus kinase signal transducer and activator of transcription (JAK-STAT) [12], and immune deficiency (IMD) pathways [13], have been recognized in *D. melanogaster*. These mechanisms regulate viral transcription and replication. The RNAi pathway affects viral replication by slicing/cleaving the viral genome [14,15,16,17]. The JAK-STAT pathways play a restricted role in only a few virus infections, whereas RNAi is active against most tested infections [12,18,19]. Genes associated with JAK-STAT and IMD pathways were reported to be silenced in sigma-virus-infected *D. melanogaster* [9], and not overexpressed, as occurs with other viruses and pathogens.

During viral infection, the host activates the immune mechanism by using pattern recognition receptors (PRRs) which detect foreign pathogen-associated molecular patterns, including viral nucleic acids and viral glycoproteins [20,21,22]. PRRs can be membrane bound, such as Toll, domeless (dome) [23], and peptidoglycan recognition protein (PGRP) [24]. Several key components instead of core RNAi mechanisms such as Dicer-2, r2d2, Argonaute-2, and piwi are responsible for initiating the antiviral response to *Drosophila* X virus (DXV), DCV, and other infectious viruses. However, host immunity may still interact with the virus and control its replication through other mechanisms. We analyzed the effect of suppressing the Toll signaling pathway on sigma virus infection, and therefore the present study aims to demonstrate individual and collective effects on viral replication in *D. melanogaster* when JAK-STAT and IMD pathways are knocked down.

## 2. Materials and Methods

### 2.1. Cells, Flies, Virus Stock, and CO_2_ Assay

*Drosophila* S2 cells were kept at 25 °C in Shields and Sang M3 insect medium (Sigma-Aldrich, St. Louis, MO, US) supplemented with yeast extract and bactopeptone according to the protocols of the *Drosophila* Genomics Resource Center (https://dgrc.bio.indiana.edu/Home), with 10% fetal bovine serum (FBS; Gibco, Grand Island, NY, US) [2]. Fly stocks were maintained in standard medium at 25 °C and 60% humidity under a 12:12 h (light:dark) photoperiod. The RC2 cell lines of *D. melanogaster*, which were or were not sigma-virus-infected, were used in this study. *Df(2R)BSC22/SM6a*, *tub-Gal4*, and *UAS-mCD8-GFP* flies were obtained from the Bloomington *Drosophila* Stock Center. A transgenic line (*tub-Gal4>UAS-mCD8-GFP*) with *green fluorescent protein* (*GFP*) gene was the progeny of *tub-Gal4* males crossed to *UAS-mCD8-GFP* females, and this line was employed to demonstrate a successful knockdown of target gene after injection of ds*gfp* (dsRNA of GFP) [25]. In order to generate the virus stock, 3000 virus-infected flies were identified via a CO_2_ assay. This works on the principal that upon exposure to high concentrations of CO_2_, uninfected (or low viral titer) flies would recover, but those with a high viral titer would be paralyzed. Therefore, as per Tsai et al. [2], flies were exposed to pure CO_2_ for approximately 30 s and kept on ice for 10 min. After 30 min, the affected flies could be separated. To release the virus from the host, the flies were frozen using liquid nitrogen and ground using 250 mL of M3^+^ medium without FBS. Fly tissues were removed through viral crude extraction by centrifugation at 800× *g* for 10 min. The suspension of the crude extraction was filtered using an NML syringe with a 0.22-μm filter (Sartorius) [26]. The extraction product containing sigma virus was collected and stored at −80 °C. For the knockdown experiments, flies were injected with 50 nL of the sigma virus using a nanoinjector (InjectMan, Eppendorf, Hamburg, Germany) [27].

### 2.2. dsRNA Preparation

The dsRNAs for *domeless* and *PGRP-LC* were synthesized using a T7 Quick High Yield RNA Synthesis Kit (New England BioLabs, Ipswich, MA, US). Templates were generated by reverse-transcription polymerase chain reaction (RT-PCR) using the primers listed in Table 1, which include a gene-specific part and a T7 promoter overhang. The final products were purified with a total RNA purification kit (GeneMark, Taipei, TW).

### 2.3. dsRNA Transfection, Virus Infection, and RNA Extraction

Approximately 2 × 10^5^ cells/well of S2 cells were seeded on a 24-well plate. The transfection mix was prepared using 5 pmol of dsRNA (ds*dome*, ds*PGRP-LC*, or dsControl), 1.5 μL of RNAiMAX transfection reagent (Life Technology, Waltham, MA, US), and M3^+^ medium to a final volume of 50 μL. The mixture was incubated for 30 min to allow the formation of a dsRNA–lipid complex, which was then added in each well and incubated at 26 °C for 1 h. The transfected S2 cells were then infected with sigma virus using 100 μL of viral extraction product. After 48 h the cells were harvested in tubes, centrifuged at 800× *g* for 10 min to remove the supernatant, and lysed using a 2-mercaptoethanol solution. RNA was isolated and purified using a total RNA purification kit (GeneMark), treated with DNase I, and finally quantified using a NanoDrop spectrophotometer (Thermo Fisher, Waltham, MA, US).

### 2.4. Gene Expression Assay Using RT-qPCR

Total RNA was reverse-transcribed using a High-Capacity cDNA Reverse Transcription Kit (Applied Biosystems, Waltham, MA, US) for RT-qPCR. A total of 500 ng of RNA sample and 0.5 µg of random primers was used.

For quantifying gene expression through RT-qPCR, a 20 μL mixture was used, containing 1 μL of cDNA, 0.5 μM forward and reverse primers, and 2× SYBR Green master mix (Applied Biosystems) under the following conditions: 94 °C for 15 s, 60 °C for 30 s, and 72°C for 30 s. The expression of actin was used as the control (Ct cutoff of 35). A list of primer sequences used in this study is given in Table 2. The relative fold changes of gene expression were calculated through the ΔΔCt method [28]. 

### 2.5. dsRNA Microinjection

Thirty virus-infected adult female flies per experiment were injected into the thorax with 30 nL of dsRNA (3 mg/mL) using a glass needle (Sutter instrument Co.) coupled to a nanoinjector (InjectMan; Eppendorf, Hamburg, Germany). Three days later, the whole bodies of flies were processed for total RNA extraction according to previous studies [29]. The obtained RNA was reverse-transcribed, and the cDNA was used for the analysis of gene expression using RT-qPCR as previously described.

### 2.6. Statistical Analysis

The *C*t values of the target genes were normalized to the *C*t values of *actin* (reference gene). The differences in expression levels of the target genes were analyzed in SPSS version using a Mann–Whitney U-test [30,31] considering *p* < 0.05 as the cutoff value for statistical significance.

## 3. Results

### 3.1. Viral Genome Replication Level Was Increased after the Knockdown of Immune Pathways in S2 Cells

**Figure 1:**
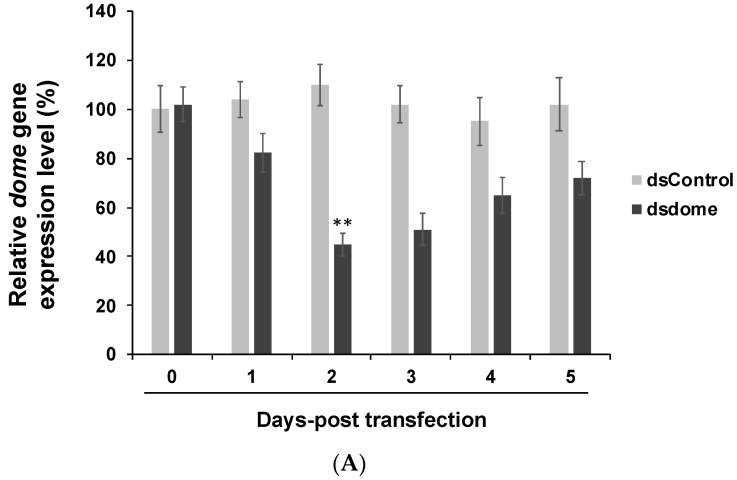

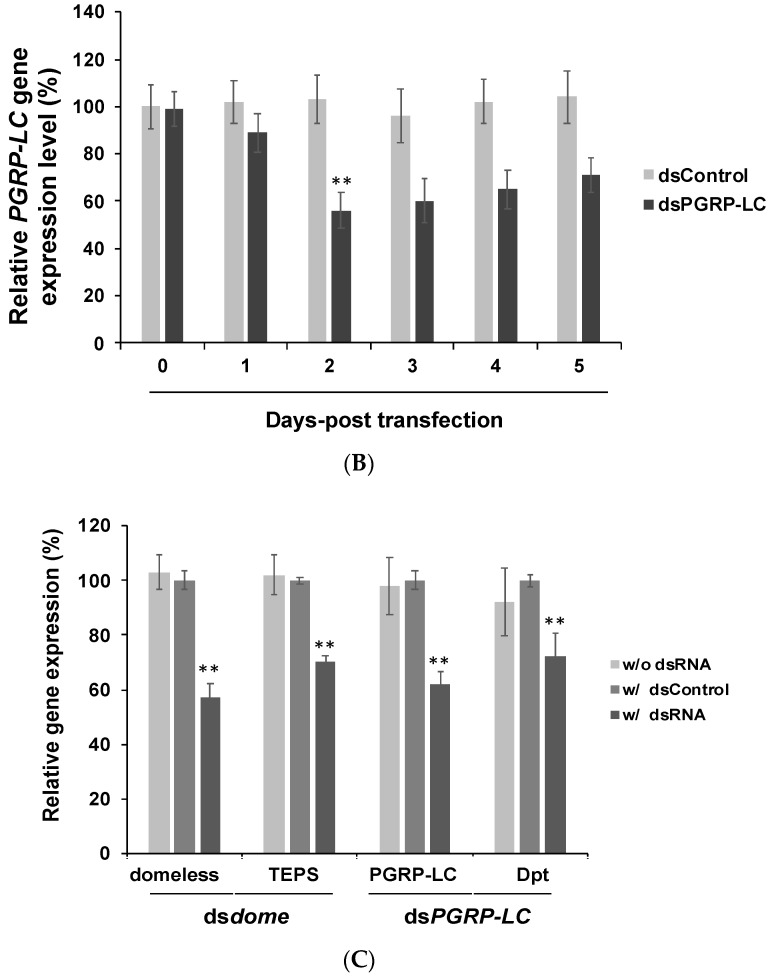
The expression of *domeless* and *PGRP-LC* decreased after being knocked down by dsRNA. (**A**) *domeless* and (**B**) *PGRP-LC* expression was at a minimum at 2 days post-transfection (dpt). *Y*-axis: relative *domeless* and *PGRP-LC* expression reading. *X*-axis: dpt. The maximum reading was set to 100, with other readings adjusted accordingly. Actin signals were used as a loading control. (**C**) The expression of antimicrobial peptide (AMP) genes detected by RT-qPCR. The downstream gene (AMP genes; *TEPS*; Dpt, *Diptericin*) expressions were decreased by silencing upstream genes. We set the dsControl group as 100% expression. Mean and SD shown, ** *p* < 0.005 one-sample *t*-test. All experiments were performed with three replicates.

We first constructed a dsRNA that specifically suppresses the upstream genes of the JAK-STAT (*domeless* gene) and IMD (*PGRP-LC* gene) pathways in S2 cells. *Drosophila* S2 cells were transfected with the dsRNA and examined each day up to 5 days post-transfection (dpt) to find the optimal knockdown time point. The suppression of *domeless* (Figure 1A) and *PGRP-LC* (Figure 1B) expression reached a maximum at 2 dpt. The suppression of upstream genes thus efficiently silenced the expression of downstream genes such as antimicrobial peptide (AMP) genes (AMP genes; *TEPS*; Dpt, *Diptericin*) (Figure 1C). 

**Figure 2:**
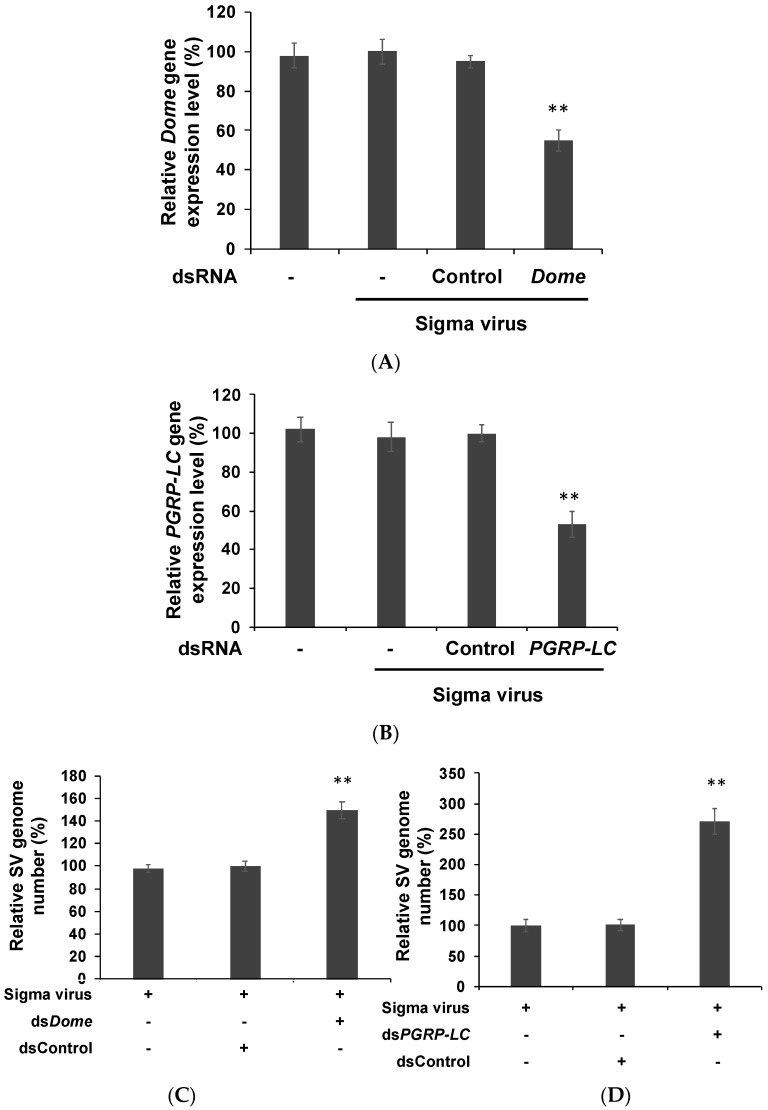
Sigma virus (SV) genome copy number increased after knocking down *PGRP-LC* and *dome*. RT-qPCR showed that dsRNA can efficiently suppress *domeless* (**A**) or *PGRP-LC* (**B**) expression in S2 cells at 2 dpt. “-”: without dsRNA transfection; “Control”: S2 cells transfected with dsControl. The viral genome replication of the sigma virus was detectable by RT-qPCR after knocking down *domeless* (**C**) or *PGRP-LC* (**D**) using dsRNA. dsControl was used as the negative control. We set the dsControl group as 100% expression. A dsRNA targeting the *GFP* gene was used as the negative control and designated dsControl. The mean and SD shown are shown. ** *p*  <  0.005, one-sample *t*-test. All experiments were performed with three biological replicates.

We knocked down the initial genes in these pathways using RNAi to examine whether there was any implication on sigma virus infectivity. As the knockdown efficiencies of dsRNA reached their maximum at 2 dpt (Figure 1), S2 cells were transfected with ds*domeless* and ds*PGRP-LC*, followed by infection with sigma virus for 48 h. We confirmed the suppression of *domeless* and *PGRP-LC* expression by subjecting *domeless* and *PGRP-LC* knockdown to RT-qPCR (Figure 2A,B). Since a previous study showed that sigma virus infects and replicates in S2 cells [32], we further studied whether *domeless* and *PGRP-LC* suppression had any effect on sigma virus replication in S2 cells. Sigma virus contains six genes, ordered 3′-*N–P–X–M–G–L*-5′. Therefore, we designed primers that covered both *N* and *P* genes in order to avoid misjudging the outcome that single gene expression difference accounted for. The viral genome copy number of the *domeless* and *PGRP-LC*-knockdown cells significantly increased (Figure 2C,D), whereas dsControl (the negative control) revealed no increase in the viral genome copy number owing to dsRNA transfection (Figure 2). Cell morphology and the number before and after dsRNA transfection were approximately the same, indicating that transfection did not cause cell death (data not shown). Our results revealed that the aforementioned suppression caused a significant increase in viral replication. A previous study showed that the expression of immunity-related genes was not altered during persistent infection; however, further suppression of the basal expression of these genes by RNAi knockdown yielded results that suggest some regulatory roles of these genes in viral replication.

### 3.2. Viral Gene Expression Increased after the Knockdown of Immune Pathways in S2 Cells

**Figure 3:**
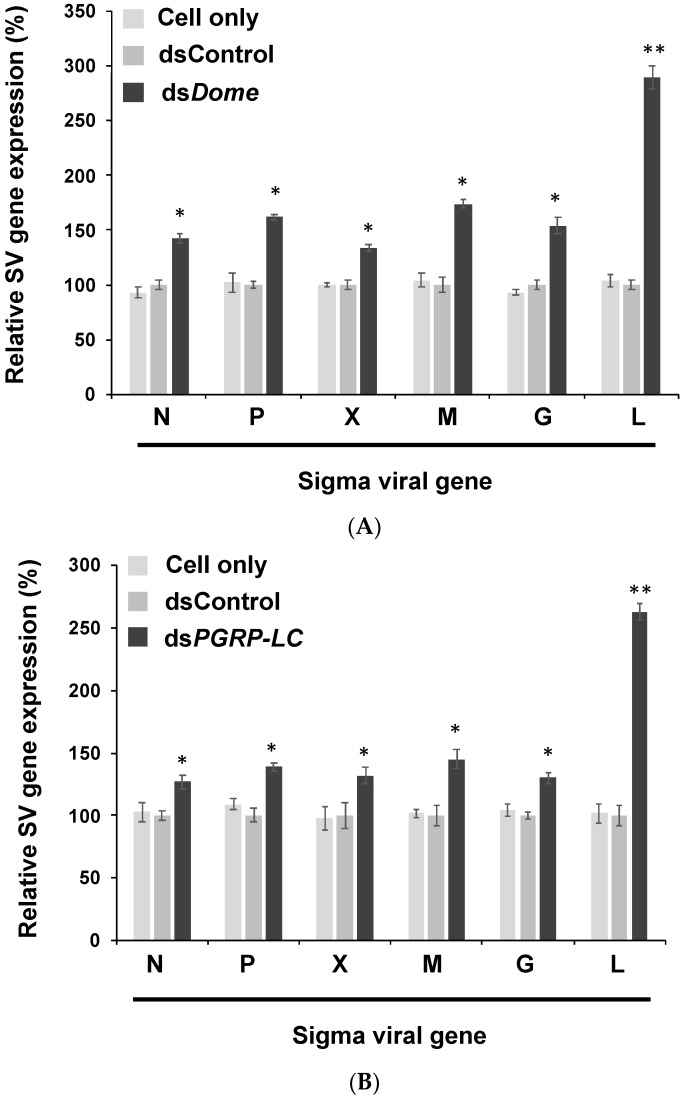
Blocking immune pathways resulted in a high level of viral gene expression. (**A**) The gene expression of the sigma virus in JAK-STAT pathway knockdown in S2 cells. The expression of all genes increased, and that of the *L* gene showed the highest increase (i.e., 4-fold). (**B**) Gene expression of sigma virus in the IMD pathway-knockdown cells. The mean and SD are shown. ** *p*  <  0.005; * *p*  <  0.05 one-sample *t*-test. All experiments were performed with three biological replicates.

To investigate whether viral genes sufficiently promote viral genome replication, we transfected S2 cells with dsRNA in order to knock down immune signaling pathways; this was followed by viral infection. After suppressing the JAK-STAT and IMD pathways, we measured the expression of the six aforementioned genes. We observed that all genes were up-regulated; in particular, the *L* gene expression increased 2.5- to 3-fold (Figure 3A,B). The protein L is responsible for replication and transcription; therefore, we proposed that the defense mechanisms governed by the JAK-STAT and IMD pathways suppress the replication and transcription of sigma virus. The knockdown of immunity-related genes in these pathways consequently increased the viral genome copy number. Most viral genes were overexpressed, and expression of the *L* gene transcripts was significantly selectively elevated. This finding indicates that the host antiviral response may control viral replication and infection through *L* gene regulation.

### 3.3. In Vivo Experiments Confirmed the Findings of in Vitro Experiments

**Figure 4:**
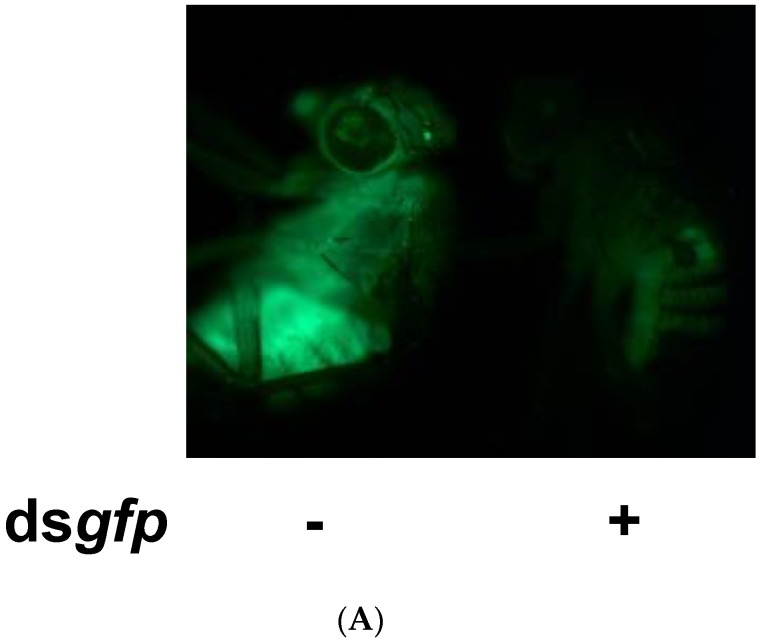

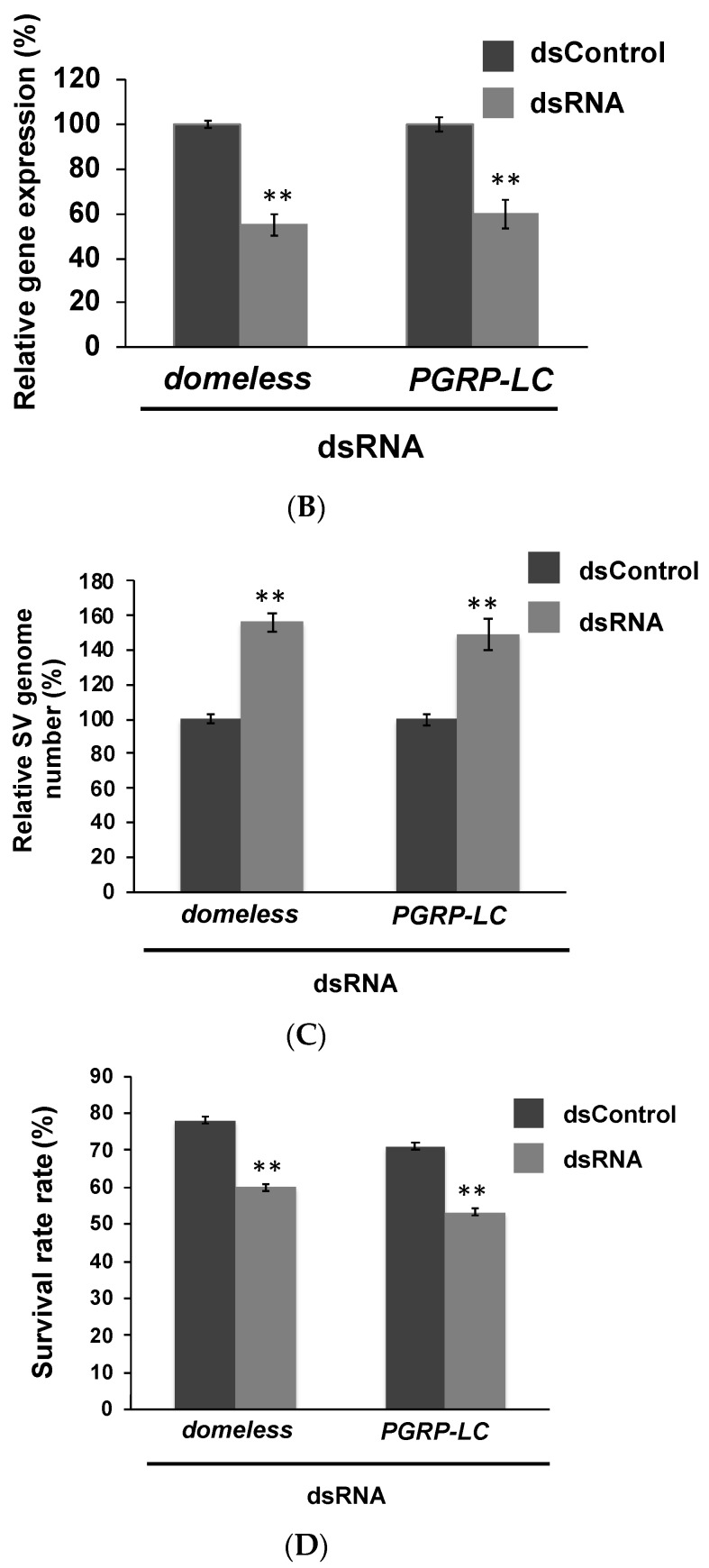

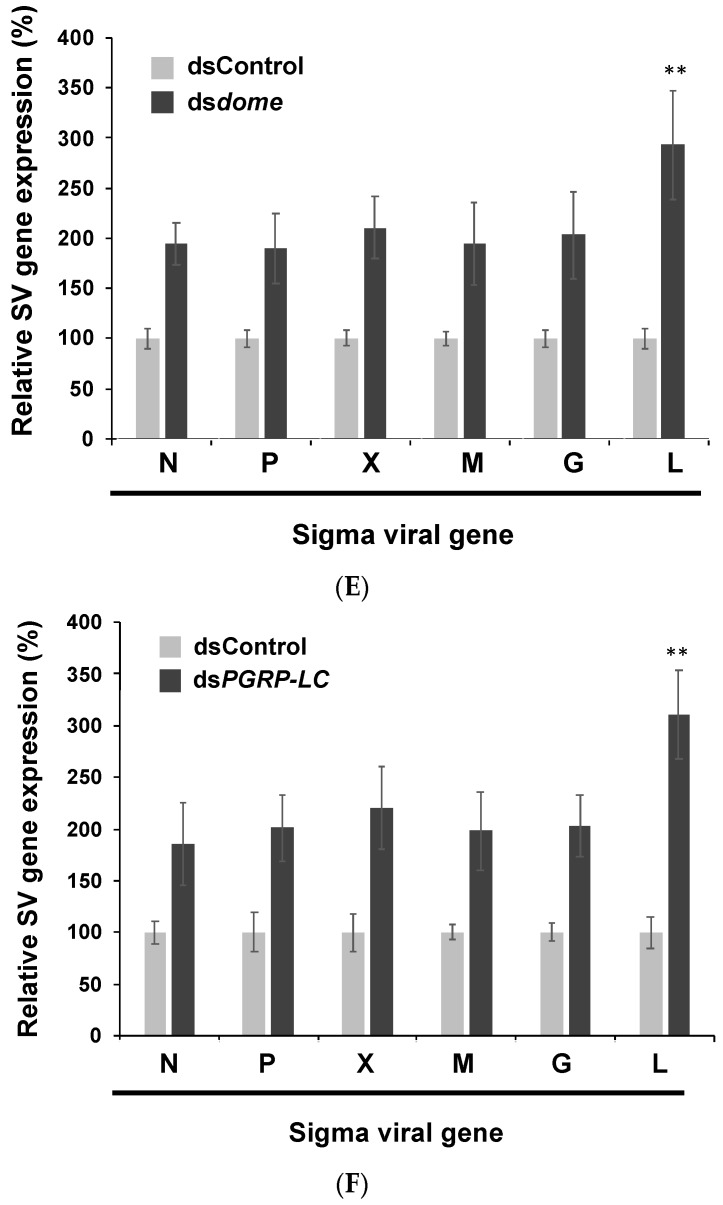
Knockdown of *domeless* and *PGRP-LC* enhanced the sigma virus replication in flies. (**A**) Green fluorescent protein (GFP) transgenic flies were injected with ds*gfp* and then reared for 3 days. The fluorescence was quenched in ds*gfp*-injected flies, indicating successful knockdown of the target gene by dsRNA injection. (**B**) RT-qPCR analysis of *domeless* and *PGRP-LC* gene expression in ds*RNA* injected flies. (**C**) The RT-qPCR analysis of sigma virus copies in sigma-virus-infected *domeless* and *PGRP-LC* knockdown and untreated flies. (**D**) The survival rate of sigma-virus-infected flies in *domeless* and *PGRP-LC*-knockdown flies upon CO_2_ exposure. (**E**,**F**) RT-qPCR analysis of sigma virus copies in sigma-virus-infected *domeless* and *PGRP-LC* -knockdown and untreated flies. We set the dsControl group as 100% expression. The mean and SD are shown. ** *p*  <  0.005, one-sample *t*-test. All experiments were performed with three replicates.

The knockdown of *Drosophila* immunity-related genes caused a high level of viral replication. Therefore, we conducted further experiments in *Drosophila* to explore whether the knockdown of host immunity genes has the same effect on persistent infection virus in vivo. ds*dome* and ds*PGRP-LC* were applied on sigma-virus-infected flies via injection. To confirm the successful knockdown of the target gene by using dsRNA, we injected ds*gfp* into GFP transgenic flies (*tub-Gal4>UAS-mCD8-GFP* fly) as the positive control [25]. The fluorescent signal in the flies decreased 3 days after injection, implying that the knockdown was achieved on day 3 (Figure 4A). We harvested sigma-virus-infected flies three days after ds*dome* or ds*PGRP-LC* injection. We examined the *domeless* and *PGRP-LC* gene expression in flies with or without dsRNA injection via RT-PCR (Figure 4B). The viral genome copy number increased approximately 1.5-fold, which was similar to the in vitro result (Figure 4C). Previous data have shown that sigma-virus-infected flies are permanently paralyzed after exposure to pure CO_2_ [7]. We therefore performed a CO_2_ assay on flies and compared the survival rate of sigma-virus-infected flies with and without *domeless* and *PGRP-LC* knockdown. The survival rate was found to be 20% lower in *domeless* and *PGRP-LC*-knockdown flies than in the wild-type flies (Figure 4D). The result indicated that the knockdown of immunity genes results in a higher replication and expression of the sigma virus and less resistance to CO_2_ treatment. We compared the transcriptional profiles of viral genes in buffer- or dsRNA-injected flies using RT-qPCR, and observed that viral gene expression was up-regulated, with the *L* gene expression significantly enhanced (Figure 4E,F). Our data suggest that immune genes protect *D. melanogaster* against sigma virus infection; this was found to be true and reproducible both in vitro and in vivo.

### 3.4. TATA-Binding Protein May Facilitate Viral Replication by Enhancing the RNA Polymerase Activity

**Figure 5:**
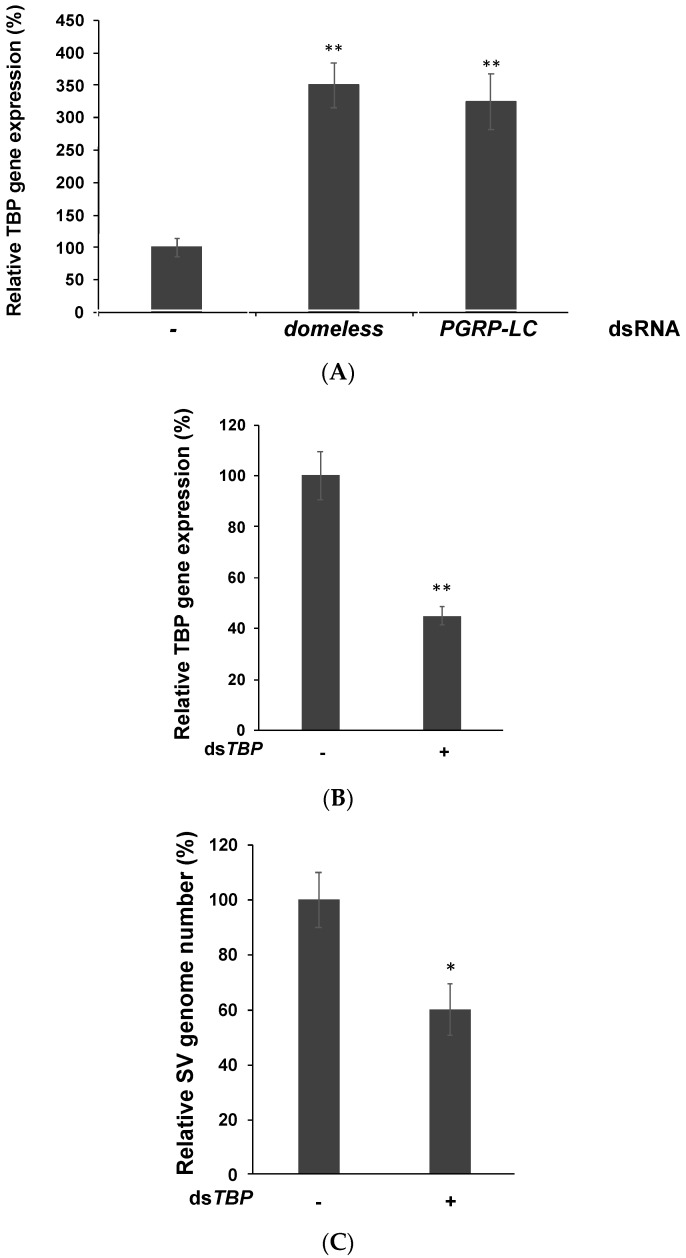
Gene expression of TATA-binding protein (TBP) increased after knocking down *domeless* and *PGRP-LC* upstream immune genes. (**A**) TBP expression increased after knocking down *domeless* and *PGRP-LC.* We set the untreated group as 100% expression. (**B**) TBP expression decreased with ds*TBP* transfected S2 cells at 2 dpt. (**C**) The RT-qPCR analysis of sigma virus copies in sigma-virus-infected *TBP*-knockdown and wild-type cells. Control treated cells were set to 100%. Mean and SD shown; ** *p*  <  0.005; * *p*  <  0.05; one-sample *t*-test. All experiments were performed with three replicates.

According to a recent study [33], in insects, TATA-binding protein (TBP) is associated with subunits of the viral RNA-dependent RNA polymerase complex (RdRp), which may indicate that TBP interacts with individual components of the viral RdRp complex for enhancing viral RNA replication. In sigma virus, the *L* gene encodes RdRp, whose main function is to regulate viral replication and translation. In our study, TBP expression markedly increased after the immune pathways were knocked down (Figure 5A). Thus, these immune pathways may suppress viral polymerase and the subsequent viral replication by reducing TBP expression. We also suppressed TBP using dsRNA and found sigma virus replication was significantly decreased (Figure 5B,C). This indicates TBP may play an important role in helping virus replicate. Currently, we are studying the relationship between TBP and sigma virus replication; these results will be published in further works.

## 4. Discussion

Viruses infect hosts to complete their life cycles. Studies have reported that the expression of host genes in the JAK-STAT and IMD pathways are unaffected in sigma-virus-infected *D. melanogaster* [9]. In our study, the expression of sigma virus genes increased after knocking down the upstream genes in these two pathways. This showed that these pathways are indeed involved in virus–host interactions (Figure 2 and Figure 3).

In *Drosophila*, the JAK-STAT pathway has been considered to be triggered in bystander cells rather than in infected cells [12]. Carpenter et al. reported that there was no difference in the gene expression of the JAK-STAT pathway between flies infected and uninfected with sigma virus [9]. However, when *domeless* was selectively knocked down in S2 cells, the gene expression and genome copy number of sigma virus increased (Figure 2). This finding revealed that the pathway regulates the replication of sigma virus. Furthermore, the expression of the six sigma virus genes increased. Both findings reveal that in infected cells, the JAK-STAT pathway affected viral replication by affecting the synthesis of viral RNA genome and proteins. We hypothesize that even though no differential gene expression was detected in factors involved in the JAK-STAT pathway, it plays an essential role in suppressing L protein expression, which in turn affects the replication of sigma virus.

The IMD pathway employs an antibacterial mechanism because its receptor PGRPs are primarily activated by bacterial peptidoglycan (PG), which is present in the membrane of Gram-negative bacteria and some Gram-positive bacteria [13,24,34]. In contrast to its well-understood antibacterial mechanism, the antiviral mechanism of the IMD pathway has been much less studied. In our study, the gene upstream of the IMD pathway, *PGRP-LC*, was selectively knocked down in S2 cells; consequently, gene expression and genome copy number of sigma virus was increased, similar to when *domeless* was knocked down. These findings reveal that the IMD pathway also affects replication of this virus in a manner similar to the JAK-STAT pathway.

Consequently, these two immune pathways were involved in the regulation of the viral polymerase in sigma virus. The production of viral RNA and protein decreased when transcriptional repressor (DR1) was knocked down in mammalian cells, suggesting that DR1 is associated with an increased viral polymerase activity [35]. Further biochemical assays revealed that viral RNA replication was suppressed in DR1-knockdown cells. According to a recent study [36], in insects, TBP is associated with subunits of the viral RNA-dependent RNA polymerase complex (RdRp), which may indicate that TBP interacts with individual components of the viral RdRp complex for enhancing viral RNA replication. In our study, TBP expression markedly increased after the immune pathways were knocked down. Thus, these immune pathways may suppress viral polymerase and the subsequent viral replication by reducing TBP expression (Figure 5). However, inhibiting the RNAi pathway did not affect TBP expression, suggesting that immune pathways regulate viral replication via a route different from that of the RNAi pathway (Figure S1). Therefore, further research is required to reveal the association between these immune genes and TBP, if present, and the involved mechanism.

The knockdown of immune pathways increases the activity of viral polymerase and replication, possibly through an increased TBP production. To date, no efficient therapy exists against many viruses, particularly against RNA viruses (e.g., HIV), because of a high mutation rate, which complicates targeted antiviral therapy [37]. A cellular immune mechanism provides nonspecific protection against viruses. Therefore, administering drugs that enhance cellular mechanisms may be an efficient method that is less subjected to viral resistance. Although the expression of all six viral genes was up-regulated when the JAK-STAT and IMD pathways were knocked down, it is worth noting that the expression of *L* was increased more than the other genes. Considering the pivotal role of *L* during viral genome transcription and replication, it can be inferred that the *Drosophila* antiviral mechanisms will target this gene. Thus, we propose that the immune system of *D. melanogaster* regulates the replication of the sigma virus by affecting the gene expression of *L*.

## 5. Conclusions

Our results showed that the viral genome copy number increased after *domeless* and *PGRP-LC* were knocked down in S2 cells. Furthermore, the expression of all six viral genes, particularly that of the *L* gene, was up-regulated. Because the *L* gene plays a crucial role in genome transcription and replication, the *Drosophila* antiviral mechanism may be activated by suppressing this gene. Thus, we propose that the immunity system of *D. melanogaster* regulates the replication of the sigma virus by affecting the *L* gene expression. Studies have shown minimal host–virus interaction in persistent infection. However, our study demonstrated that the immunity continues to affect viral replication even in persistent infection because knocking down the key components of the immune process disabled the relevant immune controls and facilitated viral expression and replication.

## Figures and Tables

**Table 1:** Primer sequences for dsRNA synthesis.

Gene Name	dsRNA Synthesis Primers
domeless (dome)	F: TAATACGACTCACTATAGGG TAACGGCAAGAGCGC
	R: TAATACGACTCACTATAGGG AGGTTCTGGCCAGGT
PGRP-LC	F: TAATACGACTCACTATAGGGG GCGGTT TCCATACGG
	R: TAATACGACTCACTATAGGGG CCATTGCTGACGCTC
GFP	F: GCTCGGGAGATCTCCTGCCTTTGGGTGTGTCTGGG
	R: CTAGACTCGAGCGGCCAACGGATCCTTCGTAGCCC
TBP	F: AATTAACCCTCACTAAAGGGAT GGACCAAATGCTAAGCCC
	R: AATTAACCCTCACTAAAGGGTACTTTCTCGCTGCCAGTCT

**Table 2:** Primer sequences used in this study.

Gene	qPCR Check Primers	Gene	qPCR Check Primers
domeless (dome)	F: ACAACAGGCGTCTTCGGATT	SV-NP	F: TAACTCGGGTGTGACAGCTC
	R: ACCCTTCAGTTTTGCCATGGT		R: CTTCGTTCATCTTCCTGGGT
PGRP-LC	F: CGCAAGGCCGTCACAGTTAC	SV-N	F: CACATGAGAAAATGCAAACAGCTT
	R: GGTTCAACGTCTTTCCGAAGAG		R: GAAAATGGAGCGAGGATCGA
Diptericin (Dpt)	F: CTATTCATTGGACTGGCTTGTGCC	SV-P	F: TCAAACCCAGAGCCAGAGATAGTAT
	R: TGGAACTGGCGACGCACTCT		R: CGCTTTTATCTGACGCTCAGGTA
TEPS	F: AACTCCGCAAACACCAAGTTGG	SV-X	F: TGGCCCCAATATTTCCTGAA
	R: CTTCAACGCTTCGTGTAACACCAC		R: GCGTCACTCCATCAGGGTTT
Actin	F: CAAAGCGCAAAAAGAACACA	SV-M	F: ACACACTCCACAGTTTACCACCAT
	R: AGAGGAGAGGGCGAGGTTAG		R: CGCCCTCCTGTCAATGAATAG
GFP	F: GTGTTCAATGCTTTGCGAGA	SV-G	F: CCATGTTTCGTTGAGCTTTCC
	R: AAAGGGCAGATTGTGTGGAC		R: CGCCTTCGTGTTCACTGAGTT
TBP	F: TAGTGGCCAATCCTGTGTACCA	SV-L	F: TTCCCTGAAGACGCCCATTA
	R: TCAGCGGAACCTGGTGTGGC		R: TGCCGCCCTCATCCAA

## Supplementary Materials

The following are available online at https://www.mdpi.com/2075-4450/10/10/339/s1, Figure S1: Gene expression of TBP increased after knocking down three upstream immune genes.
