# Supplementary material for: Identification of Regulatory Host Genes Involved in Sigma Virus Replication Using RNAi Knockdown in Drosophila
Source: Insects. 2019 Oct 11;10(10):339. doi: 10.3390/insects10100339 (PMC6835446; doi:10.3390/insects10100339)
Supplement: Supplementary file 1 [file insects-10-00339-s001.pdf]

Identification of regulatory host genes involved in sigma virus replication using RNAi knockdown in *Drosophila*

Supplementary Figure 1.

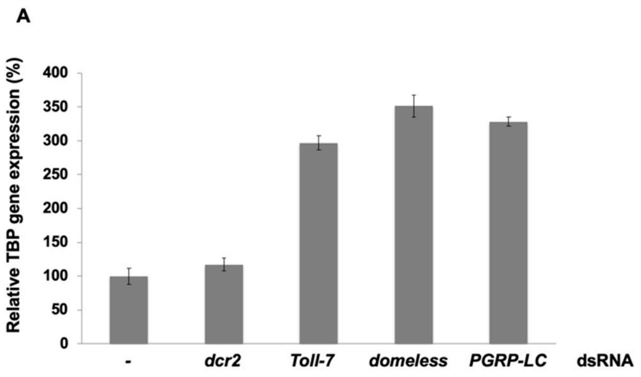

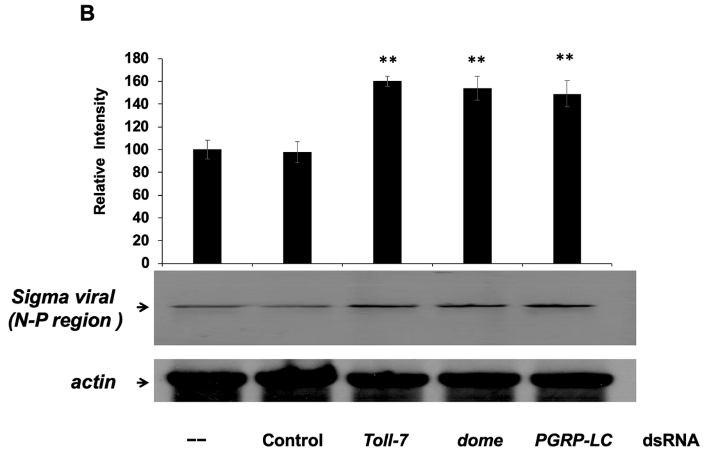

Figure S1. Gene expression of TBP increased after knocking down three upstream immune genes.

(A) TBP expression increased after knocking down *Toll-7, domeless*, and *PGRPLC* but not *dicer-2* in the transfected S2 cells. We set the untreated group as 100% expression. (B) RNA samples were subjected to Northern analysis by a full-length probe generated from the *N-P* gene fragment. The replication of viral genome in the dsRNA injected flies were probed. Actin signal were used as a loading control. We set the dsControl group as 100% expression. A dsRNA targeting *gfp* gene was used as the negative control and designated dsControl. Mean and SD shown, ** $p < 0.005$ one sample t test. All experiments were performed with three biological replicates.
